# The Eyes Don’t Have It: Lie Detection and Neuro-Linguistic Programming

**DOI:** 10.1371/journal.pone.0040259

**Published:** 2012-07-11

**Authors:** Richard Wiseman, Caroline Watt, Leanne ten Brinke, Stephen Porter, Sara-Louise Couper, Calum Rankin

**Affiliations:** 1 School of Psychology, University of Hertfordshire, Hertfordshire, United Kingdom; 2 Psychology Department, The University of Edinburgh, Edinburgh, United Kingdom; 3 Department of Psychology, University of British Columbia, Canada; University of Muenster, Germany

## Abstract

Proponents of Neuro-Linguistic Programming (NLP) claim that certain eye-movements are reliable indicators of lying. According to this notion, a person looking up to their right suggests a lie whereas looking up to their left is indicative of truth telling. Despite widespread belief in this claim, no previous research has examined its validity. In Study 1 the eye movements of participants who were lying or telling the truth were coded, but did not match the NLP patterning. In Study 2 one group of participants were told about the NLP eye-movement hypothesis whilst a second control group were not. Both groups then undertook a lie detection test. No significant differences emerged between the two groups. Study 3 involved coding the eye movements of both liars and truth tellers taking part in high profile press conferences. Once again, no significant differences were discovered. Taken together the results of the three studies fail to support the claims of NLP. The theoretical and practical implications of these findings are discussed.

## Introduction

Psychologists have carried out a great deal of research in an attempt to establish the behavioural correlates of lying [1], [2]. However, despite this impressive catalogue of work, no previous research has properly examined the validity of a notion that has received widespread acceptance among the public, namely that liars tend to exhibit a particular pattern of eye movement.

**Figure 1:**
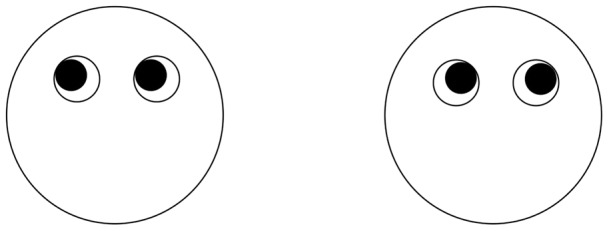
The alleged relationship between eye-movements and thinking (seen from the observer’s point of view).

Neuro-Linguistic Programming (NLP) consists of a diverse collection of psychological techniques that aim to enhance peoples’ lives [3]. An important aspect of the work involves attempting to improve people’s communication skills by teaching them about an alleged relationship between eye-movements and thought. According to this work, when right-handed people look up to their right they are likely to be visualising a ‘constructed’ (i.e., imagined) event, whilst when they look up to their left they likely to be visualising a ‘remembered’ memory (i.e., an event that has actually happened to them) (see Figure 1). In contrast, when they look to their right they are likely to be thinking about a ‘constructed’ sound, and when they look to their left they are likely to be thinking of a ‘remembered’ sound. These alleged relationships are frequently taught in NLP training courses [4], and are ubiquitous on the internet. Indeed, a Google search on the terms ‘neuro-linguistic programming’ reveals thousands of sites describing the alleged relationship, and two well known YouTube videos encouraging lie detectors to adopt this approach have received 30,000 and 60,000 views respectively.

Throughout the 1980s researchers examined many of the claims made by NLP practitioners [5], [6]. Much of this work assessed the alleged relationship between eye-movement and modality of thought, and involved recording participants’ eye-movements whilst asking them questions that encouraged to recall visual and auditory memories (e.g., ‘What colour is the front door of your house?’, ‘Can you describe the sound of your mother’s voice?’). This work consistently failed to support the claims of NLP [7], [8], [9].

Although the originators of NLP didn’t view ‘constructed’ thoughts as lies, this notion has become commonplace, leading many NLP practitioners to claim that it is possible to gain a useful insight into whether someone is lying from their eye-movements [10]. Unfortunately, very little, if any, previous work has examined the validity of this claim. Rhoads and Solomon [11] briefly refer to four experiments that allegedly demonstrated that the technique could be used to accurately classify 90% of truths and lies, but do not provide a reference for these experiments. Vrij and Lochun [12] were rightly skeptical about these alleged studies, noting that no other experiment into the psychology of lie detection has yielded this level of accuracy.

The three studies reported here provide the first experimental examination of the alleged relationship between lying and the pattern of eye-movements proposed by many NLP practitioners.

Study 1 involved filming participants lying and telling the truth, and then coding their eye movements. On the basis of the claims made by many NLP practitioners, it was predicted that participants would be significantly more likely to look up to their right when they were lying compared to when they were telling the truth, and that they would be significantly more likely to look up to their left when they were telling the truth compared to when they were lying.

## Experiment 1

### Method

#### Design

The project was approved by University of Edinburgh Psychology Research Ethics Committee (PREC), and written consent was obtained from all participants. This study employed a within design involving two conditions. In each condition participants carried out a series of actions and then took part in a videotaped interview about their behaviour. In one condition participants were asked to tell the truth, whilst in the other condition they were asked to lie. As the NLP literature does not specify what duration of eye-movements are considered informative, the study investigated both short and longer duration movements. The dependent variables were the frequency of participants’ gazes (i.e., eye-movements that lasted one second or more) and glances (eye-movements under one second in duration) to the upper right or upper left direction during the interview.

#### Participants

The 32 participants (12 male, average age 22.3, range 18–56 years) were primarily undergraduate students recruited through contacts of the experimenters. As the NLP literature suggests that the alleged relationship between lying and eye-movement is strongest in right handed people, participants were only recruited if they described themselves as right handed. Participants were told that the study concerned the psychology of lying, but were not informed that it involved studying eye-movement. Participants were not compensated for their involvement in the study.

#### Materials

Edinburgh Handedness Inventory [13]. This ten-item questionnaire asks respondents to indicate their preferred hand for using an everyday object (e.g., toothbrush). Responses are scored to give a Laterality Quotient (LQ) that has a range of possible scoring from −100 to +100. A negative score indicates a left-handed preference, and a positive score indicates a right-handed preference.

#### Procedure

Participants were tested individually. Each participant arrived at a briefing room and completed the Edinburgh Handedness Inventory. The order in which they then completed the ‘Lie’ and ‘Truth’ condition was randomly determined by a coin toss.

In the ‘Lie’ condition the participant was first given the experimenter’s mobile telephone. They were then instructed to go into a certain office, hide the telephone in their pocket or bag, and return to the briefing room. The experimenter explained that they would be taken to an interview room and filmed answering three questions: ‘What did you do inside the office?’, ‘What objects did you see in the desk drawer?’, and ‘What was the layout of the objects in the drawer?’. When asked what they did inside the office, the participant was asked to lie, and say that they opened the desk drawer and put the phone inside it. When asked what objects they saw in the drawer, the participant was asked to describe five plausible objects (‘plausible’ was defined as something small enough to fit and that might be seen in a desk drawer.) When asked to describe the layout of the objects, the participant was asked to give a fictional description. The participant was asked to be as convincing as possible throughout the interview.

The participant completed this task and was then taken to the interview room and interviewed by a second experimenter who was unaware of whether the participant was lying or telling the truth. During the interview the participant sat in front of a black background with the camera focused on the participant’s face in order that their eye-movements were clearly recorded.

In the ‘Truth’ condition the participant was given the experimenter’s mobile telephone, and instructed to go to a certain office, open the top drawer of the desk in the office, place the telephone inside the drawer, look at the other objects inside the drawer and then return to the briefing room. The experimenter explained that they would then be taken to an interview room and filmed answering three questions: ‘What did you do inside the office?’, ‘What objects did you see in the desk drawer?’, and ‘What was the layout of the objects in the drawer?’. The participant was asked to tell the truth throughout the interview. The participant completed the task and was then taken to the interview room and interviewed by a second experimenter.

The contents of the office desk drawer for each trial were randomly chosen from a pool of thirty everyday objects (e.g., stapler, apple, calculator, small umbrella, envelope).

#### Coding of interviews

**Figure 2:**
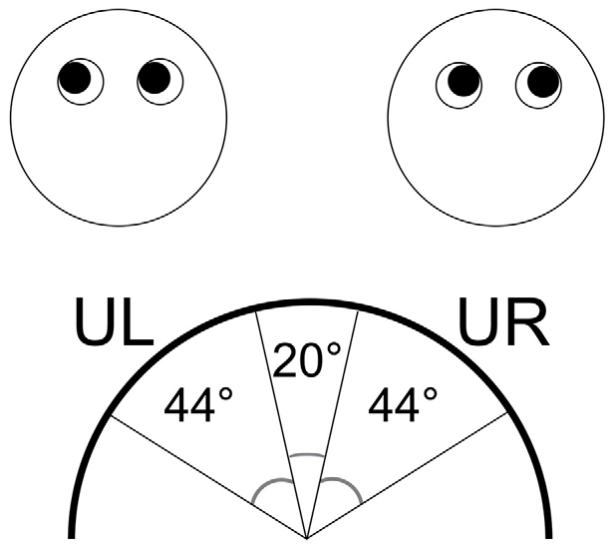
The areas of eye-movements classified as Upper Left and Upper Right (seen from the coder’s point of view).

Each of the 64 interviews (i.e., two interviews per participant) were coded by two independent raters. To ensure that the raters were not influenced by the participants’ comments during the interviews, the audio tracks were removed prior to coding. The coding involved counting the number of times the participant looked and up to the right, and up to the left, during each interview. Figure 2 depicts the two areas into which eye-movements had to fall to be coded as Upper Right (UR) or Upper Left (UL). Eye-movements in other directions (e.g. directly upwards or downwards) were not coded. To help ensure high inter-rater reliability, the raters were trained on four ‘test’ interviews that were filmed in addition to the participant interviews. One of the raters was unaware of condition allocation. The other was blind to condition allocation for 24 participants, but had conducted the interviewing for the remaining 8 participants and therefore may have been able to remember which were lies and truths. The Intraclass Correlation Coefficient for these 8 participants was 0.92 (p<0.001) vs 0.87 (p<0.001) for the 24 remaining participants. As there was little difference between these figures, there is no evidence that possible non-blind rating affected agreement between the coding.

The two raters watched each interview repeatedly at both normal and slow speed, and counted the number of times the participant looked UR for a second or longer, UL for a second or longer, UR for less than a second and UL for less than a second. Each of these values was then divided by the duration of the interview (in seconds) to create four frequency variables: UR gazes, UL gazes, UR glances, and UL glances.

For the 32 sessions, the Single Measures Intraclass Correlation Coefficient was 0.89 (p<0.001; N = 256), indicating a high level of agreement between the raters. Ratings were therefore combined for analysis.

### Results

The mean Laterality Quotient was 75.0 (SD = 22.9; range = 14.0–100.0), confirming that all participants were right-handed.

The amount of time taken for participants to give truthful answers versus deceptive answers was not significantly different (Truth mean = 43.8 seconds, SD = 19.5; Lie mean = 42.6 seconds, SD = 17.5; Related t-test (31) = 0.75, p(2-t) = 0.46).

The means and standard deviations for each of the eye-movements are shown in Table 1, along with the results of related t-tests comparing the Lie and Truth conditions. None of the analyses were significant.

**Table 1:** The mean frequencies (per second, SD in parentheses), related t-tests and p-values comparing of UL and UR gazes and glances in the Truth and Lie conditions.

	Truth condition	Lie condition	Related t-value (df = 31)	p-value(2-t)
**UL gazes**	0.012 (0.028)	0.007 (0.016)	−1.60	0.12
**UL glances**	0.040 (0.048)	0.029 (0.035)	−1.77	0.09
**UR gazes**	0.019 (0.027)	0.017 (0.023)	−0.35	0.73
**UR glances**	0.044 (0.035)	0.047 (0.033)	0.37	0.71

### Discussion

Many NLP practitioners claim that a person’s eye-movements can reveal a useful insight into whether they are lying or telling the truth. According to this notion, looking up to the right is indicative of lying whereas looking up to their left suggests that they are telling the truth. Study 1 tested both of these hypotheses but failed to find support for either notion.

It could be argued that the study lacked ecological validity because NLP practitioners do not advocate that lie detection involves the careful coding of filmed interviews, but instead promotes a more intuitive and holistic process based on the real time observation of behaviour. Study 2 tackled this issue by informing one group of participants about the eye-movements that NLP practitioners claim to be associated with lying, and then asking them to watch the interviews from Study 1 and classify each as either a lie or the truth. It was hypothesised that participants in this ‘NLP training’ group would outperform a group of control participants that had not received such training.

Second, assuming there is no relationship between the proposed patterns of eye-movements and lying, why should people come to believe that such a pattern exists? One possibility is that people are more confident in their lie detection abilities when they believe that they are following a scientific theoretical framework, such as that seemingly provided by NLP. Study 2 also addressed this question by asking participants in the ‘NLP-training’ and ‘control’ conditions to rate how confident they were about their judgements. It was hypothesised that the ‘NLP training’ group would produce significantly higher confidence levels than those in the ‘control’ group.

## Experiment 2

### Method

#### Design

The project was approved by University of Hertfordshire Psychology Research Ethics Committee, and written consent was obtained from all participants. This study employed a between-subjects design. Participants were randomly allocated to one of two groups. Participants in one of the groups were told about the pattern of eye-movement that NLP practitioners believe to be associated with lying (‘NLP training’ condition), while participants in the other group were not given this information (‘control’ condition). All participants were then asked to watch interviews from Study 1, indicate whether they thought the interviewee was lying or telling the truth, and rate how confident they were about their decision.

#### Participants

The 50 participants (16 male, average age 26.62 years, range 18–73) were recruited through contacts of the experimenters. Participants were told that the study concerned the psychology of lying, and were not compensated for their involvement in the study.

#### Materials

1. Interviews: each participant judged only the first of each pair of interviews that had been recorded for Study 1. 2. NLP training sheet: this described the patterns of eye-movement that NLP practitioners believe to be associated with lying, and provided a clear illustration of what they should be looking for during the lie detection task. 3. Response sheet: this asked participants to indicate whether they thought each interviewee was telling the truth or lying, and rate the degree of confidence in their answer on a scale between 1 (not at all confident) and 7 (very confident).

#### Procedure

All participants were tested individually. Participants were randomly assigned to either the ‘NLP training’ or ‘control’ condition. Participants in the ‘NLP training’ condition were given the NLP information sheet and asked to read it. The experimenter then answered any questions they had about the information presented on the sheet, and ensured that they understood patterns of eye-movement that NLP practitioners believe to be associated with lying. All participants were then shown the 32 video clips. After seeing each clip the participant was asked to indicate whether they believed the interviewee was lying or telling the truth, and indicate how confident they were about their decision, on the response sheet. The experimenters were blind to whether the clip being rated was the truth or a lie.

### Results

The means and standard deviations for the lie detection task and confidence ratings are shown in Table 2, along with the results of unrelated t-tests comparing the ‘NLP-training’ and ‘control’ conditions. None of the analyses was significant.

**Table 2:** Number of correct judgements (MCE = 16) in Truth and Lie conditions.

	NLP-training condition (N = 21)	Control condition (N = 29)	Unrelated t-value (48)	p-value (2-t)
**Mean correct**	16.33 (3.53)	16.59 (3.84)	−.24	.81
**Mean confidence**	4.65 (.44)	4.58 (.50)	.43	.67

### Discussion

Study 1 involved the fine-grained analysis of videotapes of liars and truth tellers, and failed to find support for claims frequently made by some NLP practitioners, namely that people tend to look up to their right when they lie and up to their left when they tell the truth. Study 2 represented a more ecologically valid test of this notion by examining the lie detection skills of people who had been informed about the alleged relationship between lying and eye movements. The study involved two groups of participants, with one group being told about the pattern of eye-movement that NLP practitioners believe to be associated with lying, while the other group were not given this information. Both groups were then asked to watch interviews from Study 1, indicate whether they thought the interviewee was lying or telling the truth, and rate how confident they were about their decision. The results revealed no difference between the accuracy levels, and confidence ratings, of the two groups and so again provided no support for the claims relating to NLP and lie detection.

The majority of psychological studies exploring lying have employed the type of ‘low-stakes’ task used in Studies 1 and 2 [14]. During these tasks people are not punished for failing to tell a convincing lie, and so participants may not be especially motivated to perform well. In everyday life this is often not the case, causing some researchers to argue that laboratory-based lie detection research lacks ecological validity. Study 3 addressed this issue by examining whether the alleged relationship between lying and eye movements emerged in a series of videos containing high stakes lies.

The study utilized a large international sample of videotapes containing footage of people making a public appeal for a missing relative [15]. In approximately half of these cases there exists overwhelming evidence suggesting that the person making the appeal was lying, whilst in the remaining cases the evidence suggests that the appeal was genuine. Previous coding of these tapes has revealed several important differences in the verbal and nonverbal behaviour of liars and truth-tellers, with, for example, liars using fewer words, more tentative words (e.g., ‘if’, ‘perhaps’, ‘maybe’), and blinking more [15].

On the basis of the claims made by many NLP practitioners, it was predicted that those lying at the press conferences would be significantly more likely to look up to their right than those telling the truth.

## Experiment 3

### Method

#### Design

The project involved coding the eye movements made by participants in two types of videos. In one set of videos there was convincing evidence that the participants were lying whilst in the other set of videos the evidence strongly suggested that they were telling the truth. As before, the study investigated both short and longer duration of eye movements to the upper right and upper left. The dependent variables were the frequency of participants’ gazes (i.e., eye-movements that lasted one second or more) and glances (eye-movements under one second in duration) to the upper right or upper left direction during the interview.

#### Videos

The video archive compiled by ten Brinke and Porter [15] contains 52 videos in which individuals make a direct public plea for the safe return of a missing relative. These videos have been gathered from news agencies in several countries, including Australia, Canada, the United Kingdom and the United States. There is compelling evidence (including, for example, possession of the murder weapon, security camera footage, the person leading police to the victim’s body, the relative being later found alive with abductor, or the relative having committed suicide) to strongly suggest that the individuals in 26 of these videos were lying and that those in the other 26 videos were telling the truth.

#### Coding

Each of the videotapes was coded by two independent raters. The coding procedure was identical to that employed in Study 1, and involved counting the number of times the participant looked up and to the right, and up to the left, during each interview. One coder analysed all of the videos whilst a second coder examined a random selection of 13 videos to assess inter-rater reliability. The inter-rater correlation coefficient was 0.85 (p<.0001; N = 52), indicating a high level of agreement between the raters. The data provided by the first rater was therefore used for the analysis.

### Results

The duration of the videos containing lies was significantly shorter than the videos containing truth telling (Truth mean = 18.37 seconds, SD = 15.70; Lie mean = 10.89 seconds, SD = 8.83; Related t-test (50) = 2.12, p(2-t) = 0.04). There were no instances of UR gazes or UL gazes. The means and standard deviations for the frequency (per second) of UR glances and UL glances are shown in Table 3, along with the results of related t-tests comparing the Lie and Truth conditions. Neither of the analyses was significant.

**Table 3:** The mean frequencies (per second, SD in parentheses), related t-tests and p-values comparing of UL and UR glances in the Lie and Truth videos.

	Truthvideos	Lie videos	Related t-value(df = 50)	p-value(2-t)
**UL glances**	0.006 (0.029)	0.015(0.05)	−0.85	0.40
**UR glances**	0.012 (0.045)	0.012 (0.054)	0.49	0.96

## Discussion

Experiment 1 tested an alleged method of lie detection promoted by many NLP practitioners. According to this notion, looking up to the observer’s left is indicative of lying, and looking up to the right signals truth telling. Participants were filmed lying and telling the truth, and coders rated each of the videos for the alleged pattern of eye-movements. The results provided no support for the existence of such patterns.

Experiment 2 involved informing one group of participants about the alleged patterns of eye movements associated with lying, and having them carry out a lie detection task. The results revealed no significant difference in accuracy between these ‘trained’ participants and those in a control condition that had not received such training. In addition, those that had received the NLP training were no more confident in their judgements. As with much laboratory-based lie detection research, it could be argued that the task used to generate the interviews lacked ecological validity. The lies were sanctioned by the experimenter, the task was relatively trivial, and there was no motivation for the participants to produce convincing falsehoods.

Experiment 3 was designed to overcome this problem and involved coding the behaviour of known liars and truth tellers in a high stakes public setting. Once again, the data did not support the claims made by NLP practitioners.

In short, all three studies provided no evidence to support the notion that the patterns of eye-movements promoted by many NLP practitioners aid lie detection. This is in line with findings from a considerable amount of previous work showing that facial clues (including eye movements) are poor indicators of deception [2]. Future research could focus on why the belief has become so widespread. Study 2 assessed the possibility that those who have been told about the claimed relationship between eye-movements and lying feel especially confident in their ability to detect deception, but this hypothesis was not supported by the data. An alternative possibility is that people believe the eye-movement/lying relationship because they are prone to illusory correlations. According to this idea, people will be likely to remember the times that the pattern predicted lying or truth-telling, and forget instances when this was not the case [16], [17]. Future work could examine this hypothesis by examining whether such matches are indeed especially memorable.

This work is the first to experimentally test the claims made by NLP practitioners about lie detection. The results provide considerable grounds to be skeptical of the notion that the proposed patterns of eye-movements provide a reliable indicator of lying. As such, it would seem irresponsible for such practitioners to continue to encourage people to make important decisions on the basis of such claims.
